# Peroxiredoxins as Potential Targets for Cardiovascular Disease

**DOI:** 10.3390/antiox10081244

**Published:** 2021-08-03

**Authors:** Se-Jin Jeong, Jong-Gil Park, Goo Taeg Oh

**Affiliations:** 1 Center for Cardiovascular Research, Washington University School of Medicine, St. Louis, MO 63110, USA; se-jin.jeong@wustl.edu; 2 Biotherapeutics Translational Research Center, Korea Research Institute of Bioscience & Biotechnology (KRIBB), 125 Gwahak-ro, Yuseong-gu, Daejeon 34141, Korea; 3 Department of Life Sciences, Heart-Immune-Brain Network Research Center, Ewha Womans University, 52 Ewhayeodae-gil, Seodaemun-gu, Seoul 03760, Korea

**Keywords:** antioxidant enzymes, aneurysm, atherosclerosis, cardiovascular diseases, hydrogen peroxide, oxidative stress, peroxiredoxins, reactive oxygen species

## Abstract

Increased oxidative stress (OS) is considered a common etiology in the pathogenesis of cardiovascular disease (CVD). Therefore, the precise regulation of reactive oxygen species (ROS) in cardiovascular cells is essential to maintain normal physiological functions. Numerous regulators of cellular homeostasis are reportedly influenced by ROS. Hydrogen peroxide (H_2_O_2_), as an endogenous ROS in aerobic cells, is a toxic substance that can induce OS. However, many studies conducted over the past two decades have provided substantial evidence that H_2_O_2_ acts as a diffusible intracellular signaling messenger. Antioxidant enzymes, including superoxide dismutases, catalase, glutathione peroxidases, and peroxiredoxins (Prdxs), maintain the balance of ROS levels against augmentation of ROS production during the pathogenesis of CVD. Especially, Prdxs are regulatory sensors of transduced intracellular signals. The intracellular abundance of Prdxs that specifically react with H_2_O_2_ act as regulatory proteins. In this review, we focus on the role of Prdxs in the regulation of ROS-induced pathological changes in the development of CVD.

## 1. Introduction

### 1.1. Oxidative Stress (OS) and Cardiovascular Disease (CVD)

CVD is caused by various heterogeneous pathophysiological mechanisms, although increased OS is considered a potential common etiology [1,2,3]. Several factors control fluctuations in the concentrations of reactive oxygen species (ROS), including the sources of ROS production and antioxidant enzymes, as the precise regulation of ROS is essential to maintain normal physiological function of cardiovascular cells [4,5,6]. The regulation of cell differentiation and growth, as well as intracellular signaling molecules, such as phosphatases and kinases, are reportedly influenced by ROS to control cellular homeostasis [7,8,9,10,11,12]. However, an overabundance of ROS due to an imbalance between the production and removal of ROS damages intracellular molecules, including DNA, lipids, and proteins, leading to cellular dysfunctions and cell death via necrosis or apoptosis [13,14]. In cells, mitochondria and enzyme systems involving nicotinamide adenine dinucleotide phosphate (NADPH) oxidases (NOXs) and xanthine oxidases are the main drivers of ROS production [15,16,17,18]. ROS constitute both nonradicals, including hydrogen peroxide (H_2_O_2_), hypochlorous acid, and ozone, and radicals, including superoxide, hydroxyl radicals, and peroxyl radicals [19]. Therefore, the actions of ROS differ according to the cell type, source, location, and concentration, which leads to a variety of physiological and pathophysiological functions [19].

**Figure 1:**
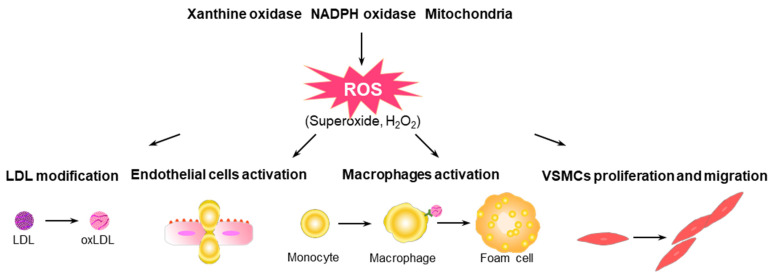
Potential roles of ROS in the progression of atherosclerosis. ROS modify LDL particles, such as oxLDLs, in the intima, which induce the expression of adhesion molecules, the release of chemokines from ECs, and the recruitment of immune cells into the intima. Monocytes recruited into the intima differentiate into macrophages that engulf oxLDLs through scavenger receptors, and macrophages create inflammatory conditions in lesions by expressing various proinflammatory cytokines, ROS, and costimulatory molecules that activate other immune cells. Excess engulfment of oxLDLs triggers the transformation of macrophages into foam cells. ROS is closely involved in the regulation of the growth, migration, extracellular matrix production, and inflammatory gene expression in VSMCs.

Atherosclerosis is a chronic vascular inflammatory disease associated with the development of CVD, which is a leading cause of mortality and morbidity worldwide [20]. Atherogenic stimuli, including dyslipidemia, hypertension, and smoking, lead to endothelial dysfunction and structural alterations to the arterial walls and trigger the accumulation of circulating apolipoprotein B (apoB)-containing lipoproteins, mainly low-density lipoproteins (LDLs), in the intima region of the arterial wall [21,22]. Structural alterations to the aorta expose subendothelial proteoglycans, which contribute to the binding of apoB to LDLs under the endothelial layer [22]. ROS modify LDL particles, such as oxidized LDLs (oxLDLs), in the intima, which induces the expression of adhesion molecules, the release of chemokines by endothelial cells (ECs), and the recruitment of immune cells into the intima [3,23]. Monocytes recruited into the intima are differentiated into macrophages, which engulf oxLDLs through scavenger receptors and create inflammatory conditions in lesions by expressing various proinflammatory cytokines and the production of ROS and costimulatory molecules that activate other immune cells [23,24]. The excess engulfment of oxLDLs triggers the transformation of macrophages into foam cells, resulting in apoptotic cell death. The apoptosis of foam cells leads to the accumulation of cellular debris and cholesterol crystals that contribute to the development of the necrotic core, thereby inducing plaque destabilization [25]. Plaque rupture or erosion causes occlusive thrombosis, leading to acute cardiovascular events, which underlie mortality due to atherosclerotic CVD [24,26]. A schematic overview of ROS-mediated pathophysiology in the development of atherosclerosis is presented in Figure 1.

Numerous studies have demonstrated an important role of ROS in the development of atherosclerosis. NOX subunit gp91phox in macrophages and NOX4 in nonphagocytic vascular cells are upregulated in human atherosclerosis, and p47phox-deficient vascular smooth muscle cells (VSMCs) produce relatively lower levels of superoxide and undergo growth factor-induced proliferation [27,28]. NOX2-mediated superoxide production reduces the bioavailability of nitric oxide (NO) and augments the development of early atherosclerotic plaque formation in apolipoprotein E knockout (*ApoE^‒/‒^*) mice [29]. The deficiency of glutathione peroxidase (Gpx)-1 exacerbates atherosclerosis and loss of heme oxygenase-1 (HO-1) accelerates the development of atherosclerosis and vascular remodeling in *ApoE^‒/‒^* mice [30,31].

**Figure 2:**
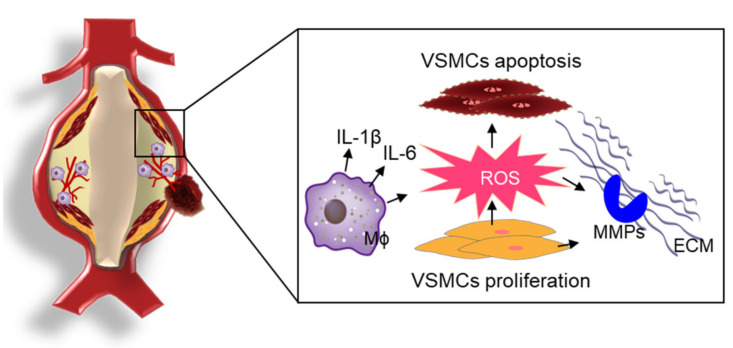
ROS-mediated the pathophysiology of AAA. In the development of AAA, ROS-induced OS is closely associated with inflammatory processes, the proliferation/migration/apoptosis of VSMCs, and the activation of MMPs for proteolytic degradation of structural proteins in the pathogenesis of AAA.

The prevalence of abdominal aortic aneurysm (AAA) ranges from 2% to 8% in men older than 65 years and the mortality rate associated with ruptured AAA varies between 85% and 90% [20,32]. The development of AAA is associated with degraded plasticity of the tunica media with the activation of various proteases and inflammation caused by the accumulation of immune cells, angiogenesis, and necrosis [33]. ROS-induced OS is closely associated with inflammatory processes and the activation of matrix metalloproteinases (MMPs), which promote proteolytic degradation of structural proteins in the pathogenesis of AAA [34]. A schematic overview of ROS-mediated pathogenesis of AAA is presented in Figure 2.

The superoxide levels and lipid peroxidation are increased and the expression of the NOX subunits p47phox and p22phox are upregulated in the aneurysmal aorta as compared with the normal aorta of human patients [35]. Inducible NO synthase in human patients with AAA, but not normal human abdominal aortas, is identified by in situ hybridization and immunohistochemical analyses [36]. The deficiency of p47phox in *ApoE^‒/‒^* mice attenuates angiotensin II (Ang II)-induced AAA formation with decreased OS, inflammation, and MMP-2 activity [37]. NOX1 deficiency reportedly prevents Ang II-induced aortic dissection with an increased expression of the tissue inhibitor of metalloproteinase-1 in mice as compared with the wild-type controls [38].

### 1.2. ROS-Mediated Pathophysiological Changes in Vascular Cells

**Figure 3:**
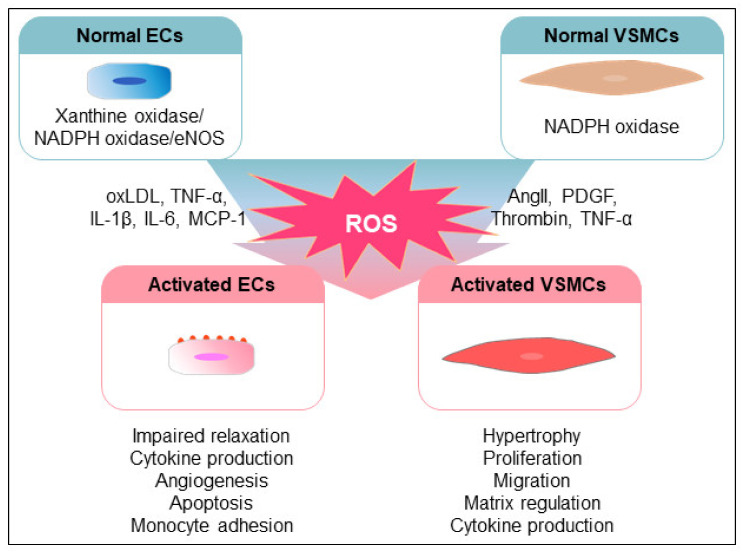
Modulation of the vascular cells by ROS. Various extracellular stimuli increase the production of ROS through ROS-generated enzyme systems in vascular cells. oxLDLs and proinflammatory cytokines induce the augmentation of ROS, which leads to the dysfunction of ECs, including impaired vasorelaxation, cytokine production, angiogenesis, apoptosis, and immune cell adhesion. Additionally, VSMCs are activated by various stimuli, such as AngII, PDGF, thrombin, and TNF-α, leading to alterations of the cellular functions, including proliferation, migration, extracellular matrix formation, and cytokine production, through increased ROS production.

OS and inflammation mainly induce dysfunction of ECs, resulting in the augmentation of vasoconstrictor and prothrombotic factors [39]. ECs modulate the vascular tone, cellular adhesion, thrombosis, smooth muscle cell proliferation, and vessel inflammation through the production of NO, prostaglandins, hyperpolarization factors, and contracting factors [40,41,42]. Therefore, the dysfunction of ECs is closely associated with an increased risk for the development of CVD and other diseases [39,43]. Endothelial nitric oxide synthase (NOS)-derived NO controls the contraction of vascular smooth muscle cells through the regulation of soluble guanylyl cyclase and cyclic guanosine monophosphate and inhibits leukocyte adhesion and platelet aggregation [42]. Cardiovascular risk factors, such as smoking, hypercholesterolemia, hypertension, and hyperglycemia, activate oxidative enzyme systems, including NOXs and uncoupled NOS, leading to the inactivation of NO by elevating superoxide levels [39,44]. The inactivation of NO induces an imbalance between EC-derived vasodilators and vasoconstrictors, resulting in endothelial dysfunction [42]. Inflammation also contributes to OS-mediated endothelial dysfunction in several human diseases, including CVD [45,46]. Proinflammatory stimuli, including oxLDL, tumor necrosis factor-alpha (TNF-α), interleukin (IL)-1β), IL-6, and monocyte chemoattractant protein-1 (MCP-1), increase the production of ROS and induce endothelial dysfunction through a variety of signaling pathways involved in activation of the nuclear factor-kappa B (NF-κB) and mitogen-activated protein kinase (MAPK) pathways [45,47,48,49]. The TNF-α-induced expression of vascular adhesion molecule-1 and intracellular adhesion molecule-1 in aortic ECs is inhibited by antioxidants and inhibitors of NF-κB and MAPK [50,51,52]. Inhibition of the oxidation of LDLs increases the expression of MCP-1 and adhesion molecules in ECs through the ROS- and NF-κB-dependent activation of lectin-like oxLDL receptor-1 [53,54]. In addition, OS and proinflammatory stimuli induce the apoptosis of ECs, resulting in atherogenesis and thrombosis, which are inhibited by superoxide dismutase (SOD), catalase, N-acetylcysteine (NAC), and vitamins [55,56]. Angiogenesis is also involved in the pathology of atherogenesis [57]. ROS regulates the migration, proliferation, and tubulogenesis of ECs by controlling intracellular signaling and angiogenic growth factor expression [43,57]. Taken together, these observations suggest that ROS is a critical factor for dysfunction and pathological signaling pathways in ECs in CVD (Figure 3).

ROS is closely involved in the regulation of the growth, migration, extracellular matrix production, and inflammatory gene expression in VSMCs [6]. The switching of VSMCs from the “contractile phenotype” to the “synthetic phenotype” is common in hypertension, atherosclerosis, aortic aneurysm, and restenosis after balloon angioplasty [58,59]. Phenotypic switching is a prerequisite for the proliferation and migration of VSMCs in the pathogenesis of CVD. Various factors, such as Ang II, platelet-derived growth factor (PDGF), and thrombin, induce ROS-dependent proliferation and the migration of VSMCs. Ang II induces the hypertrophy of VSMCs through the generation of ROS, which is inhibited by catalase and antisense p22phox [60,61]. PDGF and thrombin also induce the H_2_O_2_-dependent proliferation of VSMCs, which is inhibited by catalase, NAC, or a treatment with diphenyleneiodonium chloride [62,63]. The overexpression of catalase and peroxiredoxin (Prdx) 2 and inhibition of antioxidant production inhibit the PDGF-induced migration of VSMCs, implying that the PDGF-induced migration of VSMCs is dependent on ROS generation [11,62,64]. In addition, the production of the extracellular matrix and activation of inflammatory genes in VSMCs are regulated in a ROS-dependent manner. MMPs secreted from VSMCs are activated by ROS, and the NF-κB-mediated expression of inflammatory genes in VSMC is ROS-sensitive. The Ang II-induced expression of MCP-1 and IL-6 in VSMCs is also regulated in a ROS-dependent manner [65,66]. In addition, the TNF-α-induced MCP-1 expression in VSMCs is considered a redox-sensitive pathway [67]. Therefore, ROS-mediated alterations of the functions of VSMCs contribute to the progression of CVD (Figure 3).

Several antioxidant enzymes, including catalase, Gpxs, Prdxs, and SODs, are responsible for maintaining ROS levels against augmentation of ROS production during the pathogenesis of CVD. In this review, we focus on the role of Prdxs on the regulation of ROS-induced pathological changes in the development of CVD.

## 2. Prdxs in CVD

### 2.1. Overview of Prdxs

**Figure 4:**
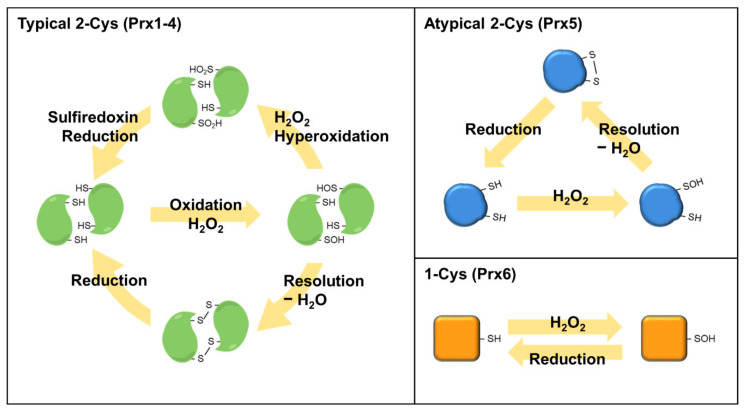
Reaction mechanisms of Prdx isoforms. The peroxidatic cysteine residue of Prdxs reacts with H_2_O_2_ to release H_2_O and form a sulfenic acid (SOH) intermediate. SOH is resolved with a second monomer to form an intermolecular disulfide bond in typical 2-Cys Prdxs (Prdx1–4). Atypical 2-Cys Prdx (Prdx5) forms an intra-subunit disulfide in the identical subunit, which is distinct from typical 2-Cys Prdxs. The 1-Cys Prdx (Prdx6) react with H_2_O_2_ to form a SOH intermediate, which forms a disulfide bond with other proteins or small thiol molecules to reduce the peroxidatic cysteine.

Prdxs, which consist of six Prdx isoforms (Prdx1–6) in mammalian cells, are conserved abundant cysteine (Cys)-based peroxidases involved in various biological functions [68,69]. Prdx1, 2, and 6 are localized in the cytosol and nucleus, while Prdx3 is restricted to the mitochondria, and Prdx5 is localized in the cytosol, mitochondria, and peroxisomes [70]. Prdxs are classified into typical 2-Cys, atypical 2-Cys, and 1-Cys Prdx subfamilies that possess diverse amino acid sequences, although all Prdxs have one conserved cysteine residue as the peroxidatic cysteine (C_P_) [68,71,72]. Typical 2-Cys Prdxs (Prdx1–4) contain two conserved cysteine residues (C_P_ and a resolving Cys, C_R_) and consist of a homodimer formed via a disulfide bond between C_P_ and C_R_ in the chain of another subunit [68,72]. Atypical 2-Cys Prdx (Prdx5) contains an intrasubunit disulfide bond between C_P_ and C_R_ in the identical subunit, which is distinct from typical 2-Cys Prdxs [68,72]. In the 1-Cys Prdx (Prdx6), C_P_ forms a disulfide bond with the C_R_ of another protein or small thiol molecules because of the lack of C_R_ in the 1-Cys Prdx [68,72]. A schematic of the catalytic mechanisms of Prdx isoforms is presented in Figure 4. By surrounding residues in the active site of Prdxs, the catalytic reactivity (rate constant, 10^6^–10^8^ M^−1^s^−1^) of C_P_ for H_2_O_2_ is markedly augmented by up to one million-fold greater than other thiol proteins [73]. H_2_O_2_, as an endogenous form of ROS in aerobic cells, is considered a toxic substance that induces OS. However, many studies conducted over the past two decades have provided substantial evidence that H_2_O_2_ acts as a diffusible intracellular signaling messenger when it is transiently produced through the activation of cell surface receptors [74,75]. Prdxs rapidly react with H_2_O_2_, peroxynitrite, and other hydroperoxides but not with other electrophiles, indicating the target-specific reactivity of Prdxs [73,76]. Intracellular Prdxs that specifically react with H_2_O_2_ act as regulatory sensors during the transduction of intracellular signals [68,72].

### 2.2. Prdx1

Prdx1 is a typical 2-Cys Prdx that is expressed in the nucleus and cytosol of several cell types. Recent studies have elucidated various functional roles of Prdx1 in CVD. One found that laminar shear stress upregulates Prdx1, which functionally decreases the shear stress-dependent H_2_O_2_ levels and maintains the redox balance in ECs [77]. It is well-known that endogenous H_2_O_2_ formation induces the release of cytokines by activated ECs and inflammation, leading to atherosclerosis [78]. By scavenging H_2_O_2_, Prdx1 suppresses the excessive activation of ECs and consequent vascular inflammation, while Prdx1 deficiency induces early atherosclerosis in *ApoE^‒/‒^* mice [79]. Moreover, another report suggested that lipophagy, the selective autophagic degradation of lipid in macrophages, is another possible protective role of Prdx1 in atherosclerosis [80]. Among the various antioxidant enzymes in macrophages, Prdx1 is highly expressed and is a major contributor to the maintenance of lipophagic flux and cholesterol homeostasis by regulating excessive H_2_O_2_ in macrophages and reducing foam cell formation and atherosclerosis [80]. While the mechanism underlying the role of Prdx1 in AAA is unknown, human studies have shown that the serum levels of Prdx1, which are increased in AAA patients [81], are positively correlated with the biomarkers plasmin-antiplasmin and myeloperoxidase and the diameter of AAA, suggesting that Prdx1 may be used as biomarker of AAA [81]. The overexpression of Prdx1 in cardiomyocytes of mice prevents transverse aortic constriction (TAC)-induced cardiac hypertrophy and heart failure, with decreased pressure overload-induced cardiac inflammation and OS via activation of the nuclear factor-erythroid 2-related factor 2/HO-1 signaling pathway [82]. In addition, the cardiomyocyte-specific expression of Prdx1 prevents doxorubicin-induced cardiotoxicity by reducing the OS and apoptosis [83]. Collectively, these results suggest that Prdx1 would be a useful target for the treatment of CVD.

### 2.3. Prdx2

Prdx2, a 2-Cys Prdx, is abundantly expressed in most cells and tissues. Although Prdx2 shares most of the features of typical 2-Cys Prdx with Prdx1, the expression patterns of Prdx2 in vascular cells appear to be relevant to its role in CVD. Prdx2 can suppress PDGF signaling by inhibiting protein tyrosine phosphatase in VSMCs and protects against PDGF-dependent neointimal thickening, which is a key feature of vascular remodeling [11]. The migration and proliferation of VSMCs during atherogenesis contribute to fibrous plaque formation, and PDGF is a strong growth factor that stimulates these pathologic processes [84]. The current evidence suggests that Prdx2 has a beneficial effect in CVD. One report demonstrated that Prdx2 is highly expressed in both vascular and immune cells, while Prdx2 deficiency accelerates atherosclerotic plaque formation and immune cell infiltration due to plaque progression, as compared with other types of antioxidant enzymes, such as Gpx1 and catalase [52]. Prdx2 expression is increased in the aneurysmal aorta of both human AAA patients and a mouse model of AAA, but the expression is relatively low in healthy humans and normal control mice, suggesting that Prdx2 is a potential biomarker of the progression of AAA [85]. In addition, Prdx2 deficiency induces the progression of AAA and most of the features of AAA tissue, including the OS, immune cell infiltration, MMP activation, elastin degradation, and aortic dilation [85]. Furthermore, Prdx2 negatively regulates H_2_O_2_ generation and thrombosis formation by platelets and VSMCs [86,87]. These findings suggest that Prdx2 may serve as a potential target for the treatment of CVD.

### 2.4. Prdx3

Prdx3 is a typical 2-Cys Prdx isoform that is exclusively localized in the mitochondria and contributes to the elimination of intracellular ROS, which is generated as a byproduct during energy production and cellular respiration. Mitochondria play central roles in various metabolic processes, including the production of cellular ATP through oxidative phosphorylation and the regulation of the intrinsic apoptosis pathway [88]. The increased mitochondrial production of ROS leads to mitochondrial DNA damage and mitochondrial dysfunction. To date, several kinds of intracellular antioxidant enzymes have been identified, especially Prdx3, which is a major antioxidant scavenger that can remove almost 90% of mitochondrial H_2_O_2_ to maintain mitochondrial homeostasis [89,90].

Prdx3 has protective roles in various cell types and ameliorates inflammation, which is highly associated with the pathogenesis of CVD. A recent study reported that the overexpression of Prdx3 protects the heart against left ventricular remodeling and failure after myocardial infarction (MI) [91]. The hallmarks of MI, such as reduced left ventricular cavity dilatation and dysfunction, myocyte hypertrophy, interstitial fibrosis, and apoptosis of the non-infarcted myocardium, are reduced in mice overexpressing Prdx3 [91]. Further, the redox state of Prdx3 significantly changes during ischemia–reperfusion heart injury [92]. The beneficial effects of Prdx-3 include maintaining cardiac function by the attenuation of the mitochondrial OS [92]. Although several studies have reported the protective roles of Prdx isoforms against atherosclerosis, the role of Prdx3 in the pathogenesis of atherosclerosis remains unclear. However, a possible mechanism of Prdx3 in atherosclerosis may be associated with mitophagy [93,94]. Mitophagy is a critical mechanism of cells to maintain metabolic homeostasis by eliminating damaged or long-lived mitochondria via autophagic degradation and has been linked with the pathogenesis of numerous human diseases, including cancers, neurodegenerative disorders, and CVD [95,96]. Although alterations to the expression of Prdx3 in mitophagy appear to be relevant, current data are insufficient to identify potential mechanisms in atherosclerosis. Nonetheless, recent studies have implicated potential roles of Prdx3 in various cell types associated with atherosclerosis [97,98].

### 2.5. Prdx4

Prdx4 is localized in the endoplasmic reticulum (ER), which is an important metabolic organelle involved in lipid and protein synthesis and transport by regulating the correct folding of proteins. The ER redox environment dictates the fate of entering proteins and the level of redox signaling mediators modulates the level of the ROS. Impairment of the ER leads to the cellular accumulation of ROS, and increased ER stress by ROS can induce apoptosis. Therefore, Prdx4 catabolizes H_2_O_2_ within the ER to provide a cytoprotective effect against the OS, acceleration of proper protein folding, and prevention of ER stress [99,100,101,102]. Prdx4 is a secretable peroxidase and, thus, a potential candidate biomarker. Prdx4 has recently been identified in the circulation of both healthy individuals and patients with peripheral artery disease (PAD) [103,104]. The overexpression of human Prdx4 in *ApoE^‒/‒^* mice highly attenuated the development of atherosclerosis by limiting the infiltration of T-lymphocytes, reducing the OS, and ameliorating necrosis [105]. In *ApoE^‒/‒^* mice with transplanted bone marrow, hematopoietic Prdx4 overexpression was sufficient to suppress the progression of atherosclerosis [105]. Therefore, Prdx4 is a potential target for treatment of CVD in the context of ER stress-induced apoptosis in the progression of atherosclerotic plaque formation [106].

### 2.6. Prdx5

Among the six Prdx isoforms, Prdx5 is the most recently discovered. Prdx5 is an atypical 2-Cys Prdx in mammals and is widely localized to the mitochondria, peroxisomes, cytosol, and nucleus. Although Prdx5 has unique features, such as localization in peroxisomes and expression as a short form and long form, which has a mitochondria targeting sequence, it has been studied less than other Prdxs [107]. Recent studies have found that Prdx5 prevents various pathological conditions by regulating the OS [108]. Prdx5 deficiency induces a susceptibility to high-fat diet-induced obesity and associated metabolic disorders [109]. Moreover, the depletion of Prdx5 significantly augmented kidney inflammation after renal ischemia–reperfusion injury [110]. Notably, extracellular Prdx5 is the strongest contributor among all Prdx isoforms to induce the expression of inflammatory cytokines through Toll-like receptor (TLR) 2 and TLR4 after a postischemic brain injury via damage-associated molecular patterns [111]. Additionally, Prdx5 can specifically bind with TLR4, and this interaction can modulate the release of proinflammatory cytokines and cell stiffening, which could be highly relevant in the inflammatory response [112]. However, several studies have indicated that intracellular Prdx5 is involved in the inhibition of inflammatory responses [113,114,115,116]. Prdx5 is constitutively expressed in human cartilage and is upregulated in osteoarthritic cartilage, suggesting that Prdx5 may have a protective function against OS in human cartilage [113]. In primary macrophages, Prdx5 expression is induced by inflammatory stimuli, such as lipopolysaccharides and interferon-γ, suggesting that Prdx5 plays a defensive role in activated macrophages [114]. However, further studies are needed to clarify the role of Prdx5 in the pathogenesis of CVD.

### 2.7. Prdx6

Prdx6 has a unique 1-Cys structure but similar functional and structural properties with other Prdx isoforms. Prdx6 has peroxidase activity via a single conserved Cys residue, short-chain organic fatty acids, and phospholipid hydroperoxides by using glutathione instead of thioredoxin as a physiological reductant. Moreover, Prdx6 is a bifunctional enzyme with glutathione peroxidase and phospholipase A2 (PLA2) activities, which characteristically responds to various extracellular stimuli [117,118]. Several recent studies have provided evidence of the role of Prdx6 in CVD. As a possible link between Prdx6 and atherosclerosis, as mentioned above, Prdx6 possesses PLA2 activity. Previous studies have demonstrated that members of the PLA2 family generate various active lipid metabolites that promote OS and inflammatory cytokine production, leading to the acceleration of inflammatory metabolic diseases, such as atherosclerosis and hyperlipidemia [119]. Additionally, Prdx6 has an important role in heart recovery following ischemia–reperfusion injury and can protect against phospholipid peroxidation-mediated membrane damage [120,121]. Prdx6-deficient mice are more susceptible to ischemia–reperfusion injury, with a reduced recovery of left ventricular function, increased MI area, and augmented apoptotic cell death [120]. The overexpression of Prdx6 in ECs prevents AngII-induced inflammation, OS, and endothelial dysfunction via inactivation of the p38 MAPK and c-Jun N-terminal kinase pathways [122]. In addition, a significant increase in plasma levels of Prdx6 has been positively correlated with endothelial dysfunction in diabetic patients with PAD [104]. Although Prdx6 has been extensively studied, more detailed studies are necessary to elucidate the role of Prdx6 in the pathogenesis of atherosclerosis. Prdx6 is reported to play a minor athero-protective role in mice with a mixed B6; 129 background [123]. Prdx6 deficiency induces higher plasma levels of lipid hydroperoxides and the oxidation of LDLs, whereas Prdx6 in transgenic mice was insufficient to protect against atherogenesis [123,124].

## 3. Conclusions and Future Perspectives

**Figure 5:**
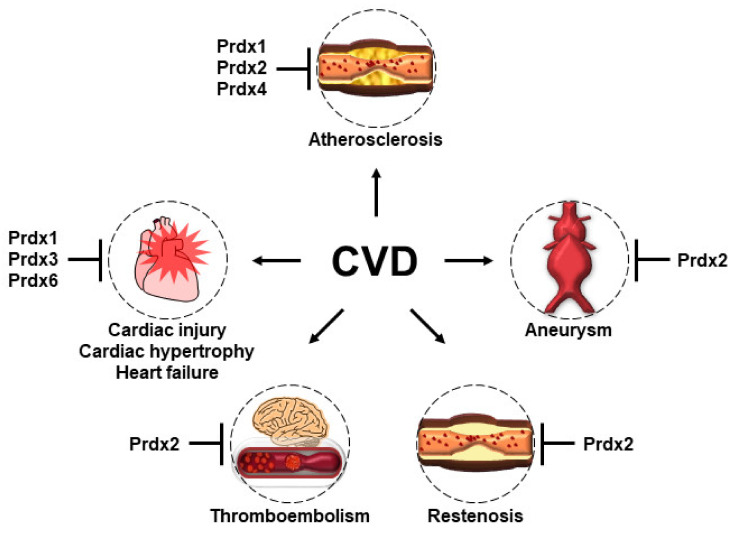
The protective roles of Prdxs in the pathogenesis of CVD. Prdx 1, 2, and 4 play protective roles in the development of atherosclerosis, although through different mechanisms. Prdx1, which is highly expressed in macrophages, maintains lipophagic flux and cholesterol homeostasis and reduces the foam cell formation and atherosclerosis. Prdx2 deficiency accelerates atherosclerotic plaque formation and immune cell infiltration. The overexpression of human Prdx4 attenuates the development of atherosclerosis by limiting the infiltration of T-lymphocytes, reducing OS, and ameliorating necrosis. Prdx2 deficiency promotes the progression and most features of AAA. Prdx2 suppresses PDGF signaling by inhibiting the protein tyrosine phosphatase in VSMCs and protects against PDGF-dependent neointimal thickening. Prdx2 negatively regulates H_2_O_2_ generation and thrombosis formation by platelets and VSMCs. The overexpression of Prdx1 in the cardiomyocytes of mice prevents TAC-induced cardiac hypertrophy and heart failure. The overexpression of Prdx3 protects the heart against left ventricular remodeling and failure after MI. Prdx6-deficient mice have increased susceptibility to ischemia–reperfusion injury.

OS, which is a common feature of CVD, is thought to occur by an imbalance in the production and removal of ROS in cardiovascular cells. Various experimental studies of dietary antioxidants and antioxidant enzymes to block the abundance of OS in CVD revealed an efficient inverse correlation. However, dietary antioxidant therapies for patients with CVD have failed in many randomized trials. To overcome the limitation of dietary antioxidants, the development of antioxidant enzymes, such as Prdxs, is a potential therapeutic strategy against CVD. Prdxs are a family of abundant thiol-dependent peroxidases, consisting of six isoforms in mammalian cells. As sensor proteins, Prdxs are appropriate antioxidant enzymes for the regulation of OS and H_2_O_2_-mediated intracellular signaling. Various experimental studies have confirmed the potential of Prdxs as a therapeutic strategy against CVD (Figure 5). Therefore, the development of derivatives or mimetics of the catalytic activity of Prdxs offers great promise for antioxidant therapy in CVD. The evolution of biotechnologies in the pharmaceutical industry could provide unique derivatives or mimetics for each Prdx isoform to develop precise medications matched to the pathophysiology of CVD.

## Abbreviations

EC	Endothelial cell
OS	Oxidative stress
CVD	Cardiovascular disease
ROS	Reactive oxygen species
NADPH	Nicotinamide adenine dinucleotide phosphate
NOX	NADPH oxidase
ApoB	Apolipoprotein B
LDL	Low-density lipoprotein
oxLDL	oxidized LDL
VSMCs	Vascular smooth muscle cells
NO	Nitric oxide
*ApoE^‒/‒^*	Apolipoprotein E knockout
Gpx	Glutathione peroxidase
HO-1	Heme oxygenase-1
AAA	Abdominal aortic aneurysm
MMPs	Matrix metalloproteinases
Ang II	Angiotensin II
TNF-α	Tumor necrosis factor-alpha
IL	Interleukin
MCP-1	Monocyte chemoattractant protein-1
NF-κB	Nuclear factor-kappa B
MAPK	Mitogen-activated protein kinase
SOD	Superoxide dismutase
NAC	N-acetylcysteine
PDGF	Platelet-derived growth factor
Prdx	Peroxiredoxin
Cys	Cysteine
C_P_	Peroxidatic cysteine
C_R_	Resolving Cys
TAC	Transverse aortic constriction
MI	Myocardial infarction
ER	Endoplasmic reticulum
PAD	Peripheral artery disease
TLR	Toll-like receptor
PLA2	Phospholipase A2
